# High-Performance Molecular Dynamics Simulations for Native Mass Spectrometry of Large Protein Complexes with the Fast Multipole Method

**DOI:** 10.1021/acs.analchem.4c03272

**Published:** 2024-09-04

**Authors:** Louise J. Persson, Cagla Sahin, Michael Landreh, Erik G. Marklund

**Affiliations:** † Department of Chemistry − BMC, Uppsala University, SE-75123 Uppsala, Sweden; ‡ Department of Microbiology, Tumor and Cell Biology, Karolinska Institutet, SE-17165 Solna, Sweden; § Department of Biology, Structural Biology and NMR Laboratory and the Linderstro̷m-Lang Centre for Protein Science, University of Copenhagen, DK-2200 Copenhagen, Denmark; ∥ Department of Cell and Molecular Biology, Uppsala University, SE-75124 Uppsala, Sweden

## Abstract

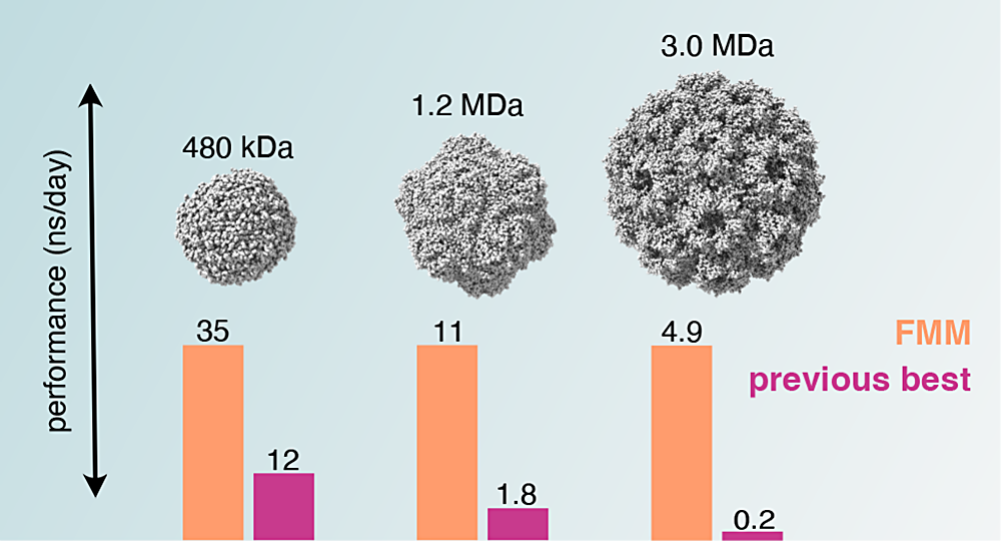

Native mass spectrometry (MS) is widely employed to study the structures and assemblies of proteins ranging from small monomers to megadalton complexes. Molecular dynamics (MD) simulation is a useful complement as it provides the spatial detail that native MS cannot offer. However, MD simulations performed in the gas phase have suffered from rapidly increasing computational costs with the system size. The primary bottleneck is the calculation of electrostatic forces, which are effective over long distances and must be explicitly computed for each atom pair, precluding efficient use of methods traditionally used to accelerate condensed-phase simulations. As a result, MD simulations have been unable to match the capacity of MS in probing large multimeric protein complexes. Here, we apply the fast multipole method (FMM) for computing the electrostatic forces, recently implemented by Kohnke et al. (*J. Chem. Theory Comput.,***2020**, *16*, 6938–6949), showing that it significantly enhances the performance of gas-phase simulations of large proteins. We assess how to achieve adequate accuracy and optimal performance with FMM, finding that it expands the accessible size range and time scales dramatically. Additionally, we simulate a 460 kDa ferritin complex over microsecond time scales, alongside complementary ion mobility (IM)-MS experiments, uncovering conformational changes that are not apparent from the IM-MS data alone.

The combination of molecular dynamics (MD) simulations and native mass spectrometry (MS) is a powerful approach for the interrogation of proteins and protein complexes.^2^ The atomistic and 3D detail achieved through MD, coupled with the ability of MS to separate and quantify different coexisting macromolecules in different states, yields comprehensive insights into the dynamic structures and assemblies adopted by proteins. MD simulations, alone or in conjunction with experiments, have provided fundamental insights into how proteins respond to the solvent-free environment of the instruments^2−8^ and into the mechanisms of electrospray ionization (ESI).^9−14^ Simulations are also increasingly being used to guide the interpretation of ion mobility (IM)-MS^15−18^ and collision-induced unfolding (CIU) experiments,^19,20^ and to aid in the development of new MS-based techniques.^21−24^

However, the rapid development of MS methods and technologies for analyzing macromolecules has not been reciprocated by gas-phase MD simulations due to a lack of efficient algorithms for computing electrostatic interactions in the absence of bulk solvent. Electrostatic forces are long-range in nature, and without a dielectric medium they exhibit a much higher effective magnitude that makes the use of traditional plain cutoff methods unsuitable for the gas phase. This is particularly true when mimicking an MS experiment, where the macromolecules are necessarily ionized and thus experience internal electrostatic repulsion, and their structures rely on a delicate balance of different forces,^25^ which would be poorly represented under an electrostatic cutoff. However, evaluating the forces acting on an atom *i* as a sum of all the pairwise interactions with Coulomb's law without a cutoff quickly becomes expensive for large systems. To see this, consider the net electrostatic force *F⃗*_Coulomb,*i*_ of all atoms *j*≠ *i* acting on atom *i*:
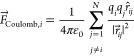
1where ε_0_ is the dielectric constant, *q*_*i*_ and *q*_*j*_ are atomic (partial) charges, *r⃗*_*ij*_ is the interatomic distance vector, and *r̂*_*ij*_ is the corresponding unit vector. Because eq 1 needs to be evaluated for all *i* ∈ {1,... *N*} atoms, it leads to  scaling. For most multimeric proteins, this degrades the performance to a level that precludes simulations over relevant time scales.^26^

Simulations of large systems in a condensed phase often employ the particle mesh Ewald (PME) method^27^ for solving the electrostatic interactions, which brings scaling down to . The method is however unsuitable for the gas phase for two reasons: (1) the approximation is correct only for systems with overall charge neutrality,^28^ which is not usually the case when simulating an electrosprayed protein, and (2) it requires the use of periodic boundary conditions (PBCs).^27^ If a small periodic box is used, artifacts can arise from interactions between the copies of the protein, but using a very large box to avoid such effects is prohibited by memory constraints and performance degradation when creating a sufficiently fine-grained mesh across the entire box. As such, it has been necessary to compute electrostatic forces by an unabridged summation of the pairwise interactions.

Recently, the fast multipole method (FMM)^29^ was adapted for computing the electrostatic interactions with the MD package GROMACS.^1,30^ Its implementation was mainly incentivized by its exceptional multinode-scaling properties, but it has additional features that might make it well-suited for simulations of proteins in vacuum: (1) it is compatible with both periodic and nonperiodic boundary conditions; (2) in contrast to cutoff-based methods, it accounts for long-range electrostatic interactions; (3) it makes no assumptions about overall charge neutrality; and (4) its scaling even exceeds that of PME at . These features have demonstrably been very beneficial for the efficient all-atom simulation of large aerosol systems spanning a volume of 2.5 × 10^6^ nm^3^.^1^ For the test systems used by Kohnke et al.,^1^ the linear scaling persisted up to system sizes of tens of millions of atoms, which augurs well for simulations of large biomolecular complexes for which the nonlinear scalings of other methods become prohibitive.

The premise of FMM (outlined in Figure 1) is to partition the system into smaller boxes by a hierarchical octree structure.^31^ The number of subdivisions *d* is set by the user, giving 8^*d*^ boxes at the deepest octree level (Figure 1A). The charge distribution of each octree box is then approximated by a truncated multipole expansion. The expression can be represented with spherical harmonics, as illustrated in Figure 1C,D. By adding higher-order terms—each of which includes multiple components that combine the order *l* and degree *m* = −*l*,...,*l*—a more detailed description is achieved. For a comprehensive explanation of this concept, refer to ref (29). The order of truncation *p*, which denotes the highest-order moments that are included in the sum, is also chosen by the user, and with increasing *p,* more terms are considered and the evaluation of the Coulomb force converges to the true value.^29^ Interactions between a point charge (an atom) and another point charge belonging to the same or to directly neighboring boxes at the deepest tree level (termed “near field”) are calculated explicitly using eq 1, whereas the multipole expansions are used for farther interactions (“far-field”; Figure 1B). Direct evaluation of interactions between an atom and a box requires a number of calculations proportional to the number of atoms in the box, whereas multipole expansion leads to only one evaluation per multipole. Moreover, empty boxes can easily be omitted to gain efficiency with sparse systems, such as molecules in a vacuum. Thereby, the number of calculations can be reduced significantly. The error arising from truncating the multipole expansion decreases with distance, which allows the use of the larger boxes at higher tree levels for far-field interactions with regions that are more separated from the point charge. As such, the farther apart a region is from an atom, the larger is the reduction in the number of calculations. Because larger systems have a higher proportion of atoms that are far apart, they will benefit the most from this approach.

**Figure 1:**
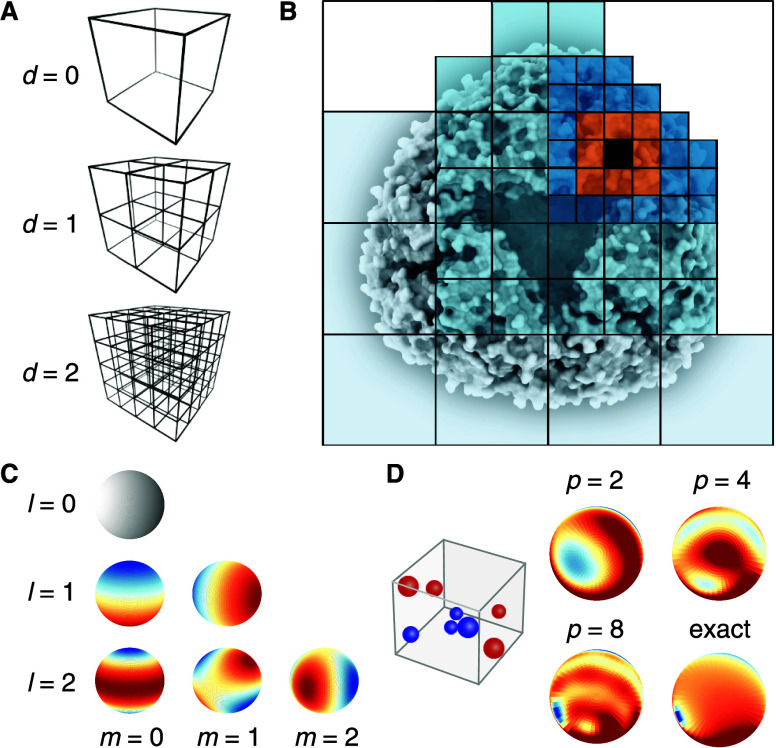
(A) Octree subdivision is shown for depth levels *d* ∈ {0, 1, 2}. (B) Principles of FMM are illustrated in 2D with *d* = 4. For a point charge belonging to the black box, the near field comprises the black and orange boxes, and blue boxes represent the far field. (C) Harmonic components for orders *l* ∈ {0, 1, 2} with non-negative values for the degree *m* (functions with negative and positive *m* are identical except their rotation around the *z*-axis). Each subplot represents the angular pattern associated with specific (*l*, *m*) combinations. (D) Comparison of numeric and analytical potentials around a set of discrete charges, with multipole expansions truncated at the annotated orders *p*. Higher-order expansions approximate the true potential increasingly accurately.

In this study, FMM is applied for the first time to simulations of large proteins in the gas phase. We evaluate how to properly use the method for simulating proteins of various sizes and shapes and provide guidelines for how to set the parameters *d* and *p* to ensure proper accuracy and optimal performance. Additionally, we apply FMM to simulate an open-pore type ferritin complex weighing 460 kDa and perform complementing IM-MS. Proteins of similar size have been simulated with all-atom MD in the gas phase before,^32^ but only on time scales of a single nanosecond, while here, we simulate for a thousand times longer. Our performance benchmarks indicate robust performance even for a 3 MDa protein, significantly exceeding the sizes of proteins that have previously been simulated in the gas phase. This will extend the range of proteins and other macromolecules that can be meaningfully simulated in the gas phase significantly and allow for computational as well as mixed studies of MS-related processes on more experimentally relevant time scales.

## Experimental Section

### Simulations with FMM

The GPU-FMM was integrated into GROMACS 2020^30^ by Kohnke et al.^1^ and was here compiled in single precision with GCC 9.3.0, CUDA 11.4, thread-MPI, and AVX2_256. The environment variables FMM_sparse and GMX_USE_GPU_BUFFER_OPS were both set to 1. Open boundaries were applied for the electrostatics but van der Waals interactions still require PBCs as the neighbor searching scheme (“*verlet*”) relies on them. The short effective range of van der Waals interactions together with the periodic image distance is sufficient to avoid self-interaction, however. Moreover, with the GROMACS FMM, the solution converges with increasing *p* only for boxes with cubic or approximately cubic geometries. Therefore, the proteins were placed in exactly cubic boxes with PBC in the *x*, *y*, and *z* directions. The minimum distance between the box and the protein was set to 3 nm to ensure enough space to account for large conformational changes that might occur.

### Simulations with Plain Coulomb Cutoffs

This study includes comparisons of the GROMACS FMM to two other methods that are commonly used for simulating proteins in the gas phase. We refer to them respectively as the “group scheme” and “pseudo-PBC” approaches. Both employ the algorithm for plain cutoffs for the Coulombic forces, but with parameters set to include the whole system, resulting in summation of all pairwise interactions using Coulomb’s law (eq 1).

The group scheme approach uses an infinite cutoff radius and open boundaries (no periodicity). This setup is compatible only with the *group* scheme for neighbor searching (hence its name), an option that was replaced by the *verlet* scheme and deprecated completely with GROMACS 5.0. At that point, GPU acceleration had not been implemented for the program, and the group scheme approach is thus restricted to CPUs. Here, we use GROMACS 4.5.7,^33^ which still has the efficient all-vs-all interaction kernels (where the actual computation of interactions take place) that were abandoned in later versions for more generic and less optimized kernels.

The pseudo-PBC approach^34^ is a workaround to make the use of GPUs possible with the current neighbor searching scheme. By using a very large periodic box and a long cutoff radius for Coulombic forces, artifacts due to the periodicity can be avoided, and newer versions of GROMACS can be used that support GPU acceleration. Specifically, a cubic periodic box with a side length of 999.9 nm and a Coulomb cutoff of 333.3 nm was used. The pseudo-PBC simulations were run with the GROMACS FMM installation, with the Coulomb-type option switched from “*FMM*” to “*cutoff*”.

### General MD Methods and Hardware

All production simulations were run on one full node of two Intel Xeon Gold 6130 CPUs, one NVIDIA T4 GPU where applicable, and one thread-MPI rank and 15 OpenMP threads. Both GROMACS installations (GROMACS FMM and GROMACS 4.5.7) employed the typical mixed-precision settings. All simulations used the OPLS-AA force field,^35^ with fourth-order LINCS constraints^36^ applied to all bonds, and virtual sites^37^ were used for hydrogens to allow for a time step of up to 5 fs. The center of mass translational and rotational velocity was removed by setting the comm-mode to “*angular*”. Production simulations used the v-rescale thermostat^38^ with a 0.2 ps time constant.

### Preparation of Systems for Benchmarking

Simulations for benchmarking the accuracy and performance of FMM and alternative methods used 14 monomeric and multimeric protein structures, retrievable from the Protein Data Bank (Table S1). The proteins were assigned net charges that had previously been reported from native MS experiments with ESI operated in the positive mode. To do this, the GROMACS command pdb2gmx was first used to predict the protonation states under neutral solution conditions. Using this as a starting point, the target net charge was then reached by protonation of the carboxylate groups of aspartates, glutamates, and C-termini. The protonation sites were chosen randomly from the residues that had a solvent-accessible surface area larger than 5 Å^2^.

Prior to production simulations, the systems were prepared with steepest-descent energy minimization and brief simulation with temperature coupling at 300 K using FMM with *d* = 0 (i.e., with explicit calculation of all pairwise interactions). For the equilibration, we employed the Berendsen thermostat^39^ and ran for 10 ps for all proteins except the largest, which required 30 ps, using a time step of 0.5 fs.

### Ferritin Simulations

MD simulations of ferritin from the archaeal species *Archaeoglobus fulgidus* were motivated by previously published IM-MS experiments.^40^ A structure is available under PDB ID 1S3Q,^41^ but that structure was not stable in the MD simulations, so a crystal structure from a humanized version of the protein (PDB ID 5LS9)^42^ was used instead, where the humanizing mutations were reverted and missing residues were added with the program Modeller.^43^ The modified 5LS9 structure was very similar to that of 1S3Q, with a root-mean-square deviation of 0.9 Å.

The protein net charge was brought to 48+, and the structure was equilibrated using the approach described under Preparation of Systems for Benchmarking above. Equilibration as well as production simulations used FMM with settings (*d*, *p*) = (3, 10). These settings were selected based on preliminary benchmarks to safeguard against potential accuracy issues while the optimal parameters were still being established. Simulations at 300 K used a time step of 5 fs, whereas at 700 K, a shorter time step of 2 fs was necessary due to the rapid atom movements. Collision cross sections (CCSs) were computed with IMPACT,^44^ using the projection approximation (PA) method^45^ and the empirical scaling factor of 1.14 from Benesch and Ruotolo^46^ to replicate the approach used in Landreh et al.^40^

### IM-MS Measurements of Ferritin

Ferritin from *A. fulgidus* with the F166H mutation was prepared as described in Deshpande et al.^47^ and exchanged into 100 mM ammonium acetate, pH 7.5, using *P*-6 Bio-Spin columns (BioRad).

Samples were introduced into the mass spectrometer using borosilicate capillaries (Thermo Fisher Scientific). Mass spectra were recorded on a Synapt G1 T-wave IM mass spectrometer (Waters) equipped with a custom pressure sleeve in the source region (MS Vision, NL). The capillary voltage was 1.5 V, the cone voltage was 100 V, and the collision voltage in the trap was raised from 10 to 100 V in 10 V steps. The source pressure was 8 mbar. Wave velocity in the IMS region was 300 m/s and wave height was 13 V. In the transfer, wave velocity was 248 m/s and wave height was 13 V. Drift gas was N_2_ with a pressure of 1.6 Torr. CCS calibrations were performed using β-galactosidase (Sigma-Aldrich) with the corresponding N_2_ CCS values.^40,46,48^ MS data were analyzed using Mass Lynx 4.1, DriftScope (Waters, Milford, MA), and PULSAR software packages.^49^

## Results and Discussion

### Accuracy

We evaluated how the accuracy of the FMM-computed Coulombic forces depends on the method-specific parameters *d* and *p* for proteins ranging from 8.6 kDa to 3.0 MDa in size, and for another set of proteins similar in size (890–980 kDa) but with different shapes. To do this, the forces were recorded over a single time step for different combinations of *d* and *p*. To find the true values for the Coulombic forces, we used GROMACS FMM without octree subdivision (*d* set to 0, making all interactions near-field), resulting in explicit summation of the pairwise interactions, as per eq 1. The error was then calculated as an average relative difference over all force components, one per each atom and spatial direction (*x*, *y*, and *z*):
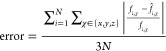
2where *f*_*i*,χ_ denotes a force component computed for atom *i* with eq 1 and *f̂*_*i*,χ_ is the value obtained with FMM.

Additionally, we computed the Coulombic forces using the group scheme approach, as described in the section Simulations with Plain Coulomb Cutoffs. This method also computes the forces explicitly and should in theory yield identical results to FMM without octree subdivision. However, when comparing the results using eq 2, they displayed a discrepancy of up to 0.016% (Figure S1). The inconsistency is likely attributed to rounding errors and other numerical effects, and we therefore use this value as a threshold for accuracy when assessing the FMM settings.

**Figure 2:**
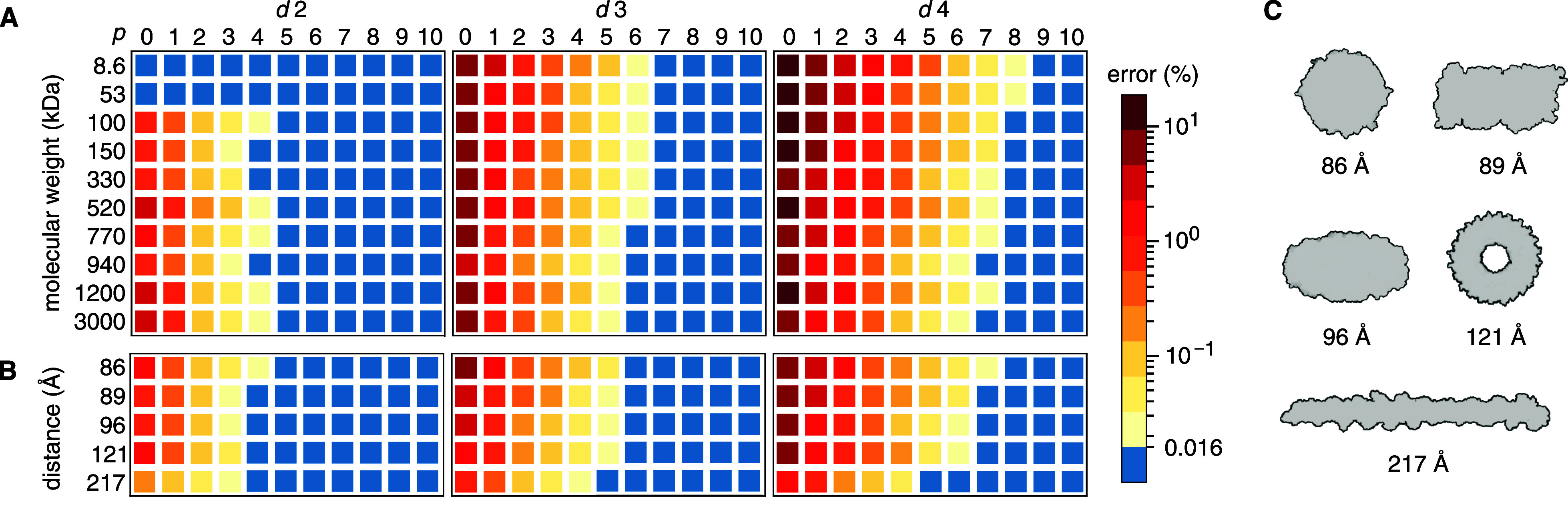
Error in the FMM-computed Coulombic forces for (A) proteins of different sizes and (B) different shapes but of similar size (890–980 kDa). Blue signifies that the error is below the accuracy threshold. Proteins compared in (B) are distinguished by their average interatomic distance, and their outlines are shown in (C). For error data in graph format, see Figure S2.

Our benchmark showed that a lower tree depth (a higher value for *d*) necessitates a higher multipole order. Evidently, the description is more sensitive to the size of the near field than to the size of the octree boxes in the far field. For all proteins, the error could be reduced below the 0.016% mark using the combinations (*d*, *p*) = (2, 5) and (3, 7) (Figure 2A,B). With *d* = 4, all proteins except for the two smallest (≤50 kDa) were also accurately simulated with *p* = 8. The error when a given multipole order is used relates to the actual size of the octree boxes. Proteins that were larger or had more extended shapes (Figure 2C) were simulated in cells with larger dimensions, making the octree boxes larger at any given tree depth. Hence, proteins with those properties reached the accuracy threshold with slightly lower values for *p*. However, little performance is to be gained from a small reduction of the multipole order (Figure S3). As such, the settings (*d*, *p*) ∈ [(2, 5), (3, 7), (4, 8)] are apt for FMM simulations of most multimeric proteins. We note that Kohnke et al.^1^ report that (*d*, *p*) = (3, 7) yields the same accuracy as PME for NaCl solutions in their benchmarks, and their errors level off at *p* between 8 and 12 just like ours (Figure S2), suggesting that our results for gas-phase proteins correspond to the case of solvated systems where accuracy is concerned.

### Performance

**Figure 3:**
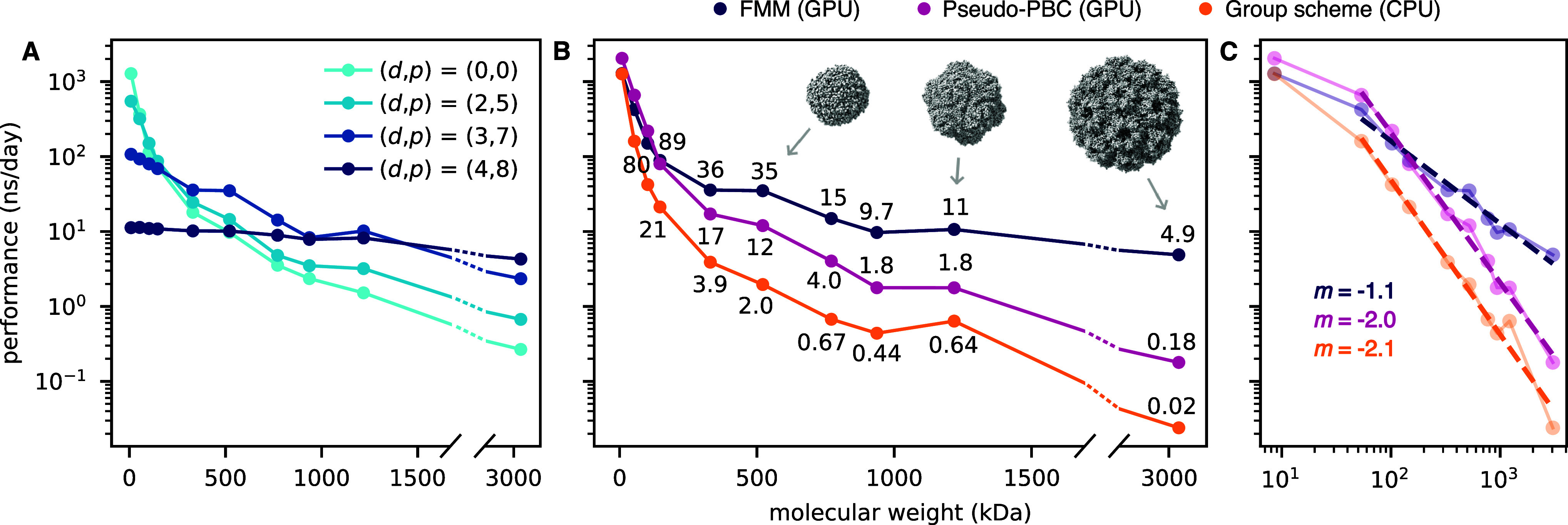
Computational performance with FMM for different protein sizes. (A) Using combinations of *d* and *p* that yield sufficient accuracy for proteins above 50 kDa. (B) Comparison of FMM using the highest-performing settings to other commonly used methods for gas-phase simulations of proteins, shown on a log–linear scale. Performance is annotated for proteins larger than 150 kDa. (C) Comparison of methods shown in log–log scale. Linear regression curves for all points except the first are shown as dashed lines with the slope (*m*) annotated.

We wanted to find out how to configure *d* and *p* in order to achieve optimal performance depending on the size of the protein. We benchmarked using simulations running for at least 15 min, using only the final half of the simulations in order to exclude any initial memory allocations and computations that are only done at startup and thus make up a negligible proportion of a long simulation. We found that the performance is strongly dependent on *d*. Figure 3A shows the performance with settings that were established to be accurate for all proteins above 50 kDa; more (*d*, *p*) combinations are shown in Figure S3. The (*d*, *p*) = (3, 7) configuration exhibited the highest performance across a broad range of proteins, from 330 kDa to approximately 1.5 MDa. Smaller proteins benefited from (*d*, *p*) = (2, 5), and larger proteins benefited from (*d*, *p*) = (4, 8). We again considered how transferable the results are to solvated systems. They can be simulated very efficiently with other methods such as PME, giving even better performance in most cases except for extraordinarily large systems.^50^ The parameter values for optimal performance with FMM will moreover be implementation dependent. For example, Ohno et al.^51^ use local octrees connected in a higher-level tree with arbitrary subdivision dimensions, complicating the comparison with our *d*-values. Andoh et al.^52^ present another implementation that employs octree-like structures but do not systematically explore the impact of depth on performance, using *d* = 6 throughout. We conclude that optimal *d*-values for solvated systems are implementation-dependent and might not be the same as those reported here for gas-phase proteins.

Next, we compared the FMM performance to the two methods that are commonly used for gas-phase simulations of proteins, the group scheme approach and the pseudo-PBC approach described in Simulations with Plain Coulomb Cutoffs (Figure 3B). Here, we used the (*d*, *p*) values giving the highest performance with errors below the 0.016% threshold for each protein. We found that FMM is the fastest method for simulating gas-phase proteins above 150 kDa, whereas pseudo-PBC outperformed FMM for smaller proteins, albeit by a small margin. As expected, the performance gain is larger with larger proteins; with the largest system that was evaluated, a 3.0 MDa capsid from the cowpea chlorotic mottle virus, FMM reached an impressive 27-fold acceleration compared to the pseudo-PBC method. Compared with the group scheme approach, FMM achieved a 200-fold acceleration. The difference can be explained not only by the different scaling behaviors of the two algorithms but also by the fact that the group scheme runs on CPUs only, which very likely limits its performance compared to the other algorithms that utilize GPUs.

Plotting the performance in nanoseconds/day against the number of atoms *N* in the log–log scale allowed us to assess the scaling in quantitative terms. The larger the value of *d*, the larger the mass required to reach the linear regime, as is apparent from comparing the different parameter combinations for FMM (Figure S4). The combinations (*d*, *p*) = (0, 0), (2, 5), and (3, 7) approached straight lines, whereas for *d* = 4, it is difficult to say with certainty whether that had happened even at 3 MDa. With (*d*, *p*) = (0, 0) and (2, 5), the lines had slopes of approximately −1.8 and −1.6, suggesting worse-than-expected linear scaling. While puzzling at the first glance, this nonoptimal performance can probably be explained by the computational overhead from keeping the octrees and multipoles up to date slowing down the too small systems where the benefits from using FMM are minor, or where the octree depth is too small for larger systems so that an unnecessary fraction of the interactions are calculated in the near-field regime. Interestingly, when considering the best-performing (*d*, *p*) combination for each protein, we get a line with slope −1.1, corresponding to a defacto scaling of approximately  (Figure 3C). A similar slope is achieved with (*d*, *p*) = (3, 7), which can be assumed to reflect the observation that most of our test systems had the highest performance with this combination of parameters. In contrast, the group scheme approach and the pseudo-PBC approach gave rise to slopes of −2.1 and −2.0. As such, both the group scheme and the pseudo-PBC approaches scaled approximately like the expected , whereas FMM, with the best-performing parameters, displayed linear scaling with no sign of deviation even for multi-MDa systems.

### Ferritin Changes Shape without Collapsing upon Activation

The performance enhancement granted by FMM for large gaseous proteins was leveraged in applied simulations of a 460 kDa complex. The protein, a ferritin from *A. fulgidus*, forms homo-24-mers shaped like spherical cages with four large pores. Hollow assemblies have been understudied by IM-MS, leading us to investigate how a protein with surface-accessible cavities reacts to ionization and activation. In order to keep a close connection with IM-MS experiments, we chose to monitor the CCS of the ferritin complex as that is a quantity that can be inferred from the IM data. The CCS is the effective size of an ion as it passes through a buffer gas, where large ions make more collisions with the gas than small ones do and thus take longer time to reach the end of the IM cell. The structure of an ion will affect the number and nature of the collisions so that different conformations of a protein might have different CCSs. As such, the CCS can be used with molecular modeling to assess the structures of molecules and their aggregates.^45^

**Figure 4:**
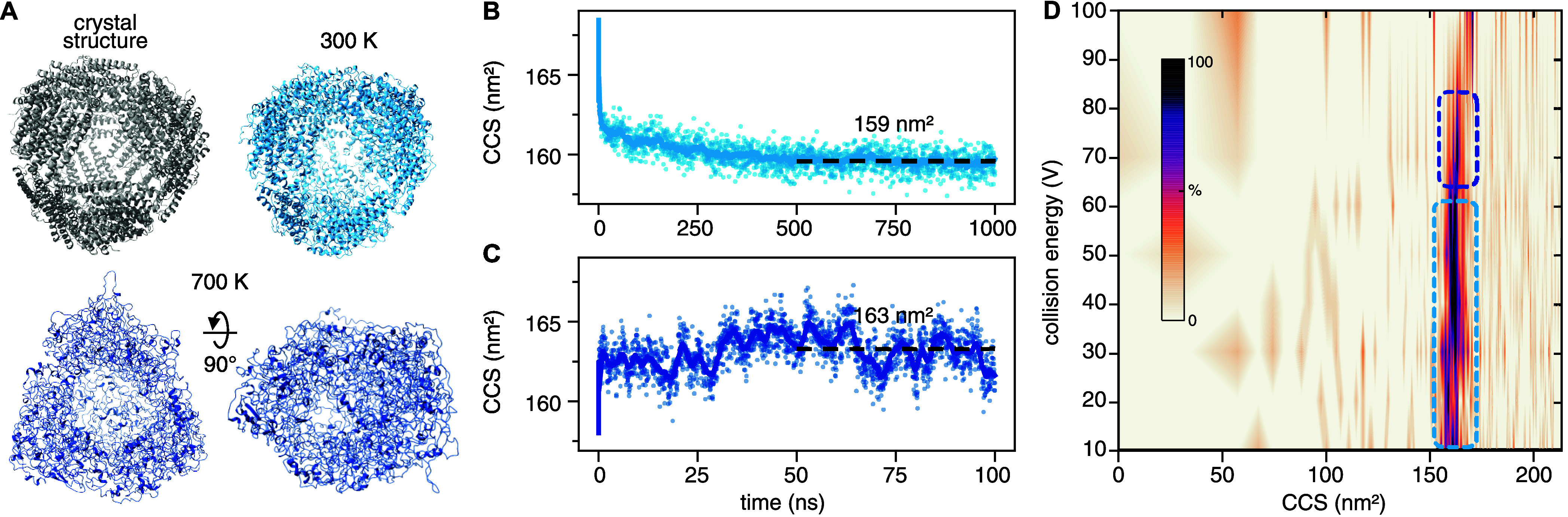
MD and IM-MS data of ferritin. (A) Crystal structure and final simulation structures at the indicated temperatures. Theoretical CCS during MD simulations at 300 K (B) and 700 K (C), with dashed lines showing the average value over the final half of the trajectory. (D) Experimental CIU fingerprint for the 48+ charge state. Encircled regions connect the experimental CCS to the computational models with corresponding colors in (A–C).

First, we simulated ferritin in vacuum at 300 K for 1 μs, at the 48+ charge state based on earlier experiments.^40^ Under these conditions, the structure remained largely intact and the CCS stabilized at 159 nm^2^ (Figure 4A, B), which represents a vacuum compaction of 6.5%. This is on par with what is typically seen for gas-phase proteins.^7,16,23^ In an attempt to induce larger structural changes, we mimicked a CIU experiment, where proteins are subjected to a range of collision voltages, while their CCS is monitored with IM-MS. A common approach to do this with MD is to use increased temperature to represent the increased energy arising from the collisions.^16,19,20^ Here, we elevated the temperature to 700 K and simulated ferritin for an additional 100 ns, resulting in a significant loss of secondary structure and of the spherical shape (Figure 4A). But instead of collapsing, the complex was stretched out to resemble a tetrahedron. Interestingly, the CCS remained largely unperturbed by the change in structure and fluctuated between 160 and 166 nm^2^ (Figure 4C).

We note that the CCSs of the spherical and tetrahedral models differ by only 2.5%. To validate these models, we recorded IM-MS data of *A. fulgidus* ferritin at different trap voltages using a Waters Synapt G1 ion mobility mass spectrometer, which had been modified with a pressure sleeve in the first pumping stage to facilitate soft desolvation (MS Vision, NL). We find that at collision voltages between 10 and 60 V, the protein maintains a constant CCS of approximately 162 nm^2^ (Table S2 and Figure S5), although the 48+ charge state corresponds well with the MD simulations at 159 nm^2^ (Figure 4D). At 70–80 V, we observe a small increase in CCS ranging from 1 to 3% from low to high charge states. This change is compatible with the small CCS increase observed in models at higher temperatures, although a comprehensive comparison would require considering contributions from multiple charge states. At 90 V, the mass spectra show a sudden increase in free ferritin monomers and a near-complete loss of the signal of the 24-mer, indicating a relatively well-defined threshold for dissociation. Interestingly, we previously measured the CCS of *A. fulgidus* ferritin on an unmodified Synapt G1 and observed a 12% compaction compared to the crystal structure.^40^ It is tempting to speculate that the gentler desolvation in the modified instrument facilitates the evaporation of solvent from the inside of the hollow ferritin structure, whereas harsher desolvation conditions lead to structural collapse below the dissociation threshold.

Comparing IM data with those of MD or other molecular models comes with several challenges. First, the traveling wave instrument used here requires calibration against proteins in the appropriate mass range,^48^ but other factors can affect mobility too.^53^ Here, the hollow ferritin complex will differ from the globular proteins typically used for calibration, making accurate and absolute CCSs somewhat difficult to obtain without using, for example, drift tube instruments. The CCSs calculated from the structures can also suffer from systematic errors. Here, we use the PA method because it is unparalleled in terms of speed, which is particularly important for large macromolecular structures. It is however on the less advanced end of the spectrum of CCS calculation methods, and it lacks sensitivity to certain geometries, at least in extreme cases.^54^ In addition, it must be recognized that the classical force fields used for simulations like ours are not parametrized for gas-phase proteins at 700 K and might be less accurate models of such systems. As such, one must be open to the possibility that the remarkable correspondence between the CCSs in our simulated and experimental CIU might be coincidental. Even so, the striking similarity between the relative CCS increases seen at high activation with both MD and IM-MS is reassuring, suggesting that the structural changes we see in the simulations also take place in the experiments. Our results underscore the value of complementing experimental IM-MS data with MD simulations, as conformations that are drastically different can otherwise be overlooked due to overlapping CCS values. In practice, this can mean that structural changes that bring little or no change to the CCS will go unnoticed if one relies solely on CIU experiments. A similar observation has been made for dimeric insulin, which underwent structural changes when adapting to vacuum conditions in long MD simulations (albeit with PME electrostatics), but without significant change to the CCS.^55^ Additionally, we note that ferritin took several hundreds of nanoseconds to fully equilibrate to the vacuum environment, which highlights the necessity of using long simulation times with large proteins in the gas phase for this type of study.

## Conclusions

For MD simulations of proteins above 150 kDa in the gas phase, FMM is faster than other methods at computing the electrostatic forces. In contrast to the quadratic scaling of other approaches, FMM scales linearly with the number of atoms, yielding good performance even for proteins in the MDa range. The acceleration ensures that multimeric protein complexes can be simulated with atomistic MD over meaningful time scales, allowing MD simulations to match the size range that can be analyzed with native MS, and taking great strides toward accommodating time scales relevant for the experiments. Here, simulations of a 460 kDa ferritin cage were run with FMM for an entire microsecond at room temperature, and an additional 100 ns at elevated temperature, revealing that such time scales can be required for large proteins to fully equilibrate to vacuum conditions. Comparison of the resulting models to data obtained by IM-MS showcased that very different conformations can have overlapping CCS values, highlighting the value of corroborating experimental CCSs with computations.

We hope that this work will promote the adoption of FMM and inspire its application alongside various experimental techniques that require gas-phase macromolecules. For example, ESI is an excellent technique for delivering protein complexes for single-particle imaging with X-ray free-electron lasers,^56,57^ where single gas-phase particles are exposed to X-rays bright and short enough to yield diffraction patterns from which the structures can be determined.^58^ Combining with IM-MS additionally enables selection of specific subpopulations from a heterogeneous mixture of proteins or proteoforms.^59^ Atomistic MD simulations have not yet been fully able to complement this technique, with the 800-kDa GroEL complex being the smallest particle detected so far,^60^ whereas the majority of MD investigations have been restricted to proteins of a few kDa at most. With the performance offered by FMM, MD simulations can now play a role in the development of this method. Native MS has also recently proven to be a powerful means to separate and deposit intact protein complexes on grids for cryo-electron microscopy, allowing the determination of high-resolution structures.^24^ Here too, long simulations of large macromolecular complexes have great potential to advance the method further and to stake out new paths toward novel experiments.

Lastly, we see great potential in FMM-supported MD for advancing native MS and IM, in addition to its value when used to model specific macromolecular systems. To date, there are a number of factors that remain unknown or poorly understood in (IM-)MS, including the protonation of basic groups on the protein surface, conformational transitions upon activation, and more. By extending the size range and time scales on which proteins can be simulated in the gas phase, FMM can enable new hypotheses to be tested. We foresee that this will have a notable impact on our understanding of MS fundamentals. The juxtaposition of MD simulations and experiments opens new avenues to improve the physical models and algorithms underpinning the simulations, which will enable yet new experimental comparisons and so forth. As such, the dramatic capacity increase that FMM brings to gas-phase MD is an important component in future iterations of combined experiments and computations that can drive the development of theory, methods, and technology for MS.

## Supporting Information Available

Detailed information about proteins used for benchmarks; extended accuracy and performance data; and extended IM-MS data (PDF)
