# Supplementary material for: High-Performance Molecular Dynamics Simulations for Native Mass Spectrometry of Large Protein Complexes with the Fast Multipole Method
Source: Anal Chem. 2024 Sep 4;96(37):15023–30. doi: 10.1021/acs.analchem.4c03272 (PMC11411496; doi:10.1021/acs.analchem.4c03272)
Supplement: Supplementary file 1 — ac4c03272_si_001.pdf [file ac4c03272_si_001.pdf]

Supporting Information

High-performance molecular dynamics simulations for native mass spectrometry of large protein complexes with the fast multipole method

Louise J. Persson[†], Cagla Sahin[‡,¶], Michael Landreh[§,‡], and Erik G. Marklund[†]

[†]Department of Chemistry – BMC, Uppsala University, SE-75123, Uppsala, Sweden
[‡]Department of Microbiology, Tumor and Cell Biology, Karolinska Institutet, SE-17165 Solna, Sweden
[¶]Department of Biology, Structural Biology and NMR Laboratory and the Linderstrøm-Lang Centre for Protein Science, University of Copenhagen, DK-2200 Copenhagen, Denmark
[§]Department of Cell and Molecular Biology, Uppsala University, SE-75124 Uppsala, Sweden

August 16, 2024

Table S1: Protein structures used for benchmarks. Virtual sites are included in the atom count.

**protein**	**PDB ID**	**size (kDa)**	**net charge**	**# atoms**
ubiquitin[a]	1UBQ	8.6	7+	1,400
transthyretin (4mer)[a]	1F41	50	15+	7,700
concanavalin (4mer)[a]	1AZD	100	21+	15,500
alcohol dehydrogenase (4mer)[a]	2HCY	150	24+	22,600
glutamate dehydrogenase (6mer)[a]	1NQT	330	40+	50,100
ferritin (24mer)[a]	7A6A	480	51+	71,600
GroEL (14mer)[a]	1SS8	770	68+	121,700
PA200/20S (29mer)[b]	6KWY	890	77+	136,400
TRiC (16mer)[b]	6KS6	930	77+	145,300
Hsp60/Hsp10 (28mer)[a,b]	6HT7	940	77+	148,800
muscle thin filament (23mer)[b]	5MVA	960	77+	145,300
needle complex (48mer)[b]	2Y9J	980	77+	148,800
STNV coat protein (60mer)[a]	4V4M	1200	90+	186,700
cowpea chlorotic mottle virus (180mer)[a]	1CWP	3000	160+	477,900

[a] Used in comparison of protein sizes; [b] Used in comparison of protein shapes.

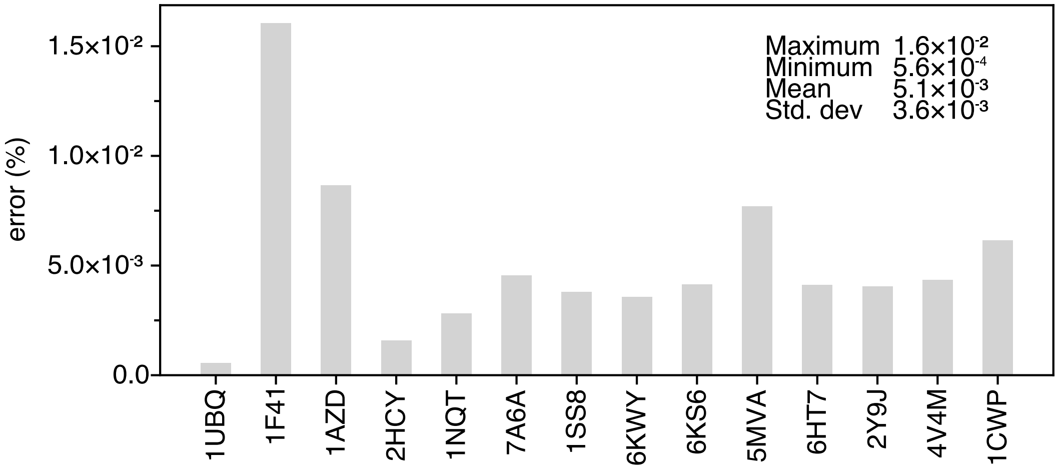

Figure S1: Difference in Coulombic forces computed with FMM using $d = 0$ and the group scheme approach. Proteins are labeled by their PDB IDs and are sorted from lowest to highest molecular weight.

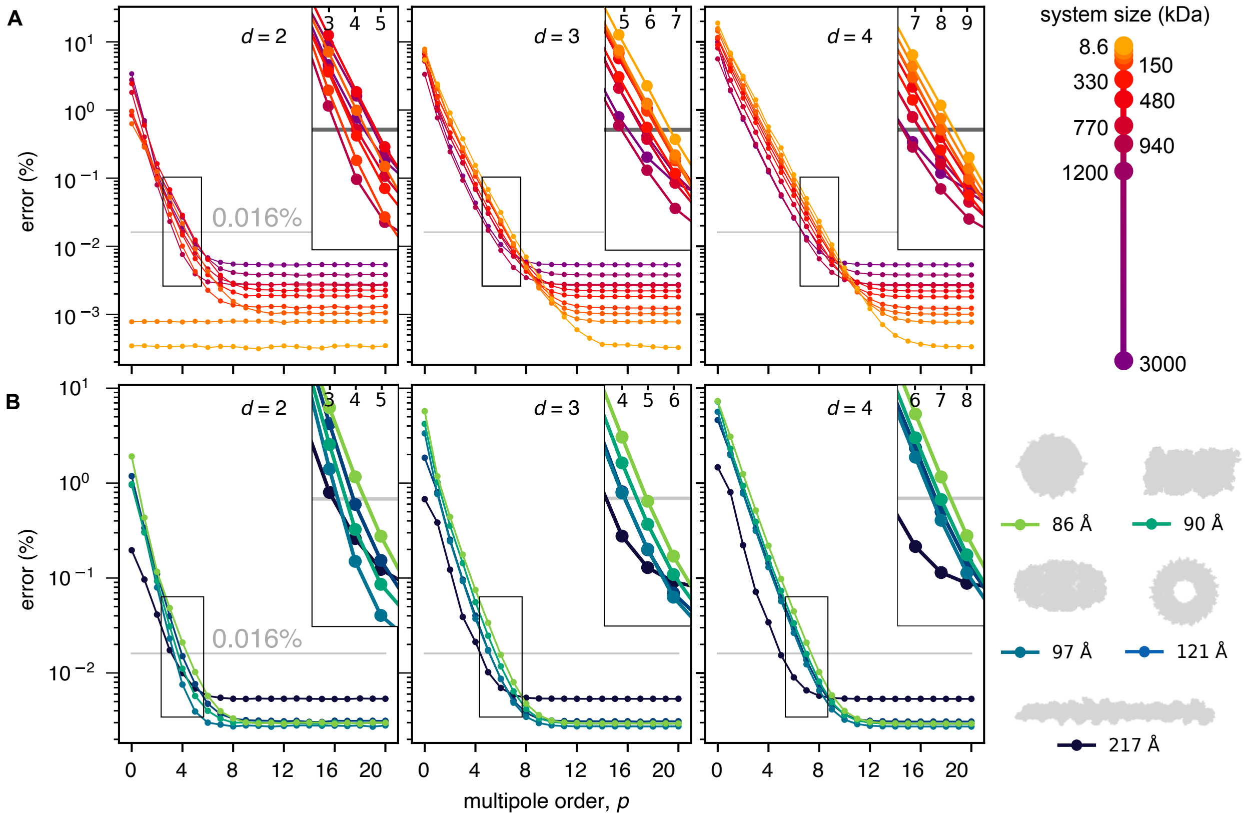

Figure S2: Error in the FMM-computed Coulombic forces for proteins of different sizes (A) and different shapes (B). Zoom-ins of where the accuracy threshold (grey line) is traversed. Outlines of proteins with different shapes and their average inter-atomic distances shown in B.

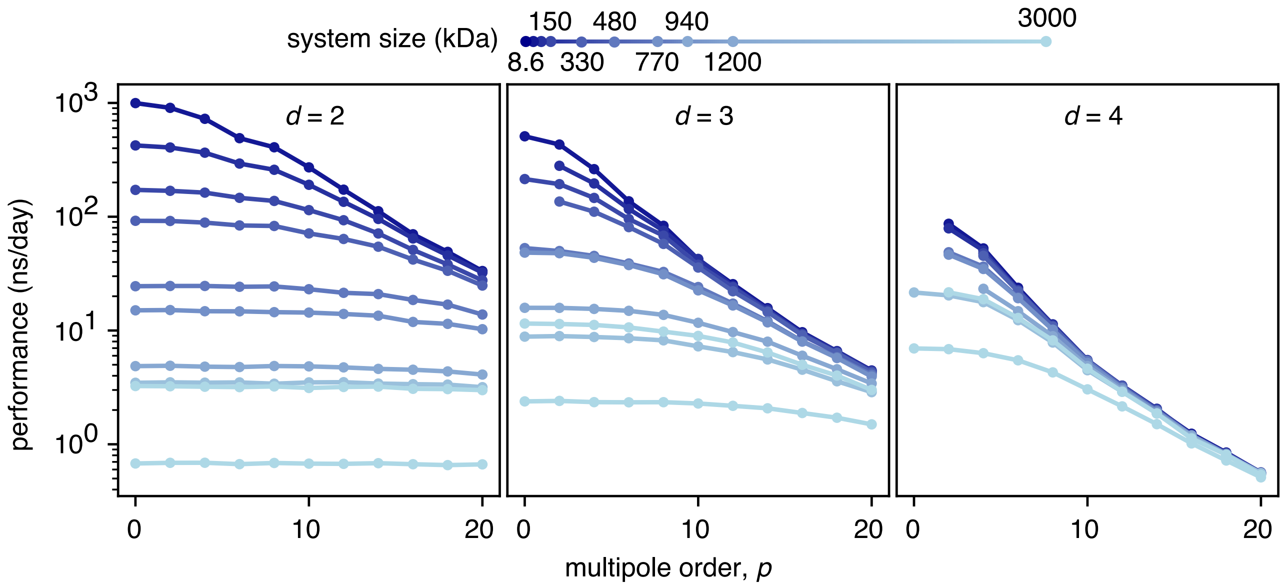

Figure S3: Computational performance with FMM. Showing the complete data set with all combinations of d and p that were tested. Some combinations were too inaccurate to generate a stable simulation, resulting in missing data points.

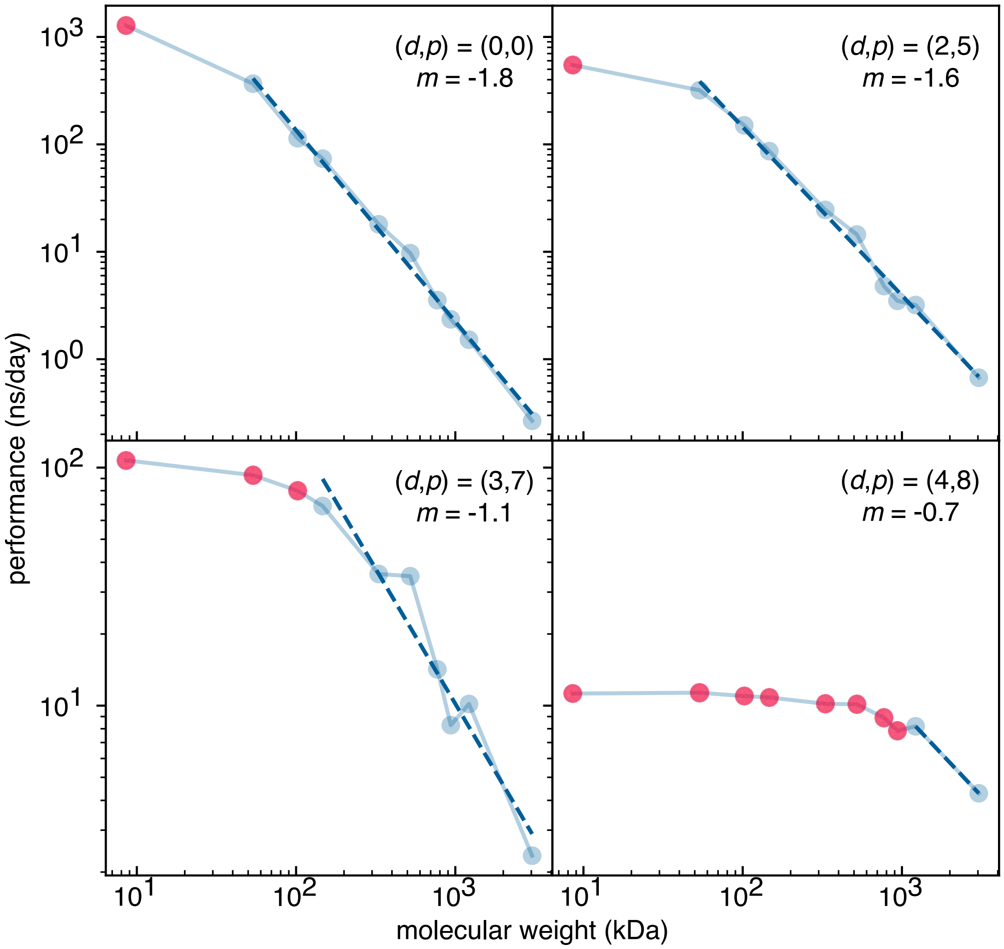

Figure S4: Computational performance against system size with the accurate combinations of d and p, shown in log-log scale. Dashed lines represent linear regressions, with slopes (m) annotated. Data points marked with red are not included in the regression as linear scaling is not anticipated for small proteins that do not adequately fill the simulation box.

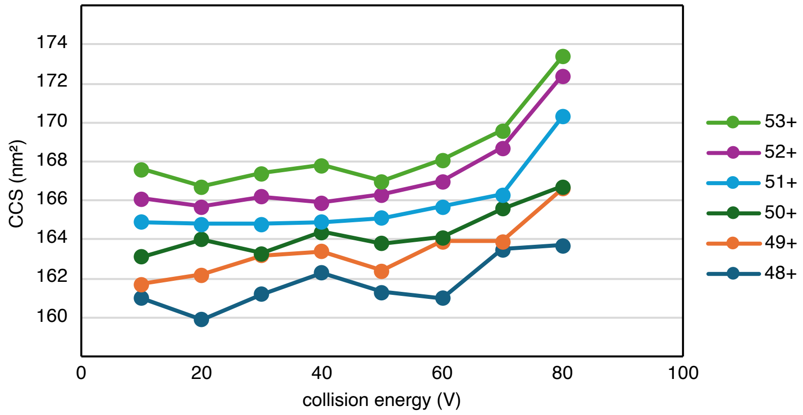

Figure S5: IM-MS measurements of ferritin, taken from Table 2.

Table S2: IM-MS measurements of ferritin.

		48+	49+	50+	51+	52+	53+	Avg.	Std. dev.
**CCS (nm^2)**	**10 V**	161.0	161.7	163.1	164.9	166.1	167.6	164.1	2.579
	**20 V**	159.9	162.2	164.0	164.8	165.7	166.7	163.9	2.481
	**30 V**	161.2	163.2	163.3	164.8	166.2	167.4	164.4	2.248
	**40 V**	162.3	163.4	164.4	164.9	165.9	167.8	164.8	1.928
	**50 V**	161.3	162.4	163.8	165.1	166.3	167.0	164.3	2.227
	**60 V**	161.0	163.9	164.1	165.7	167.0	168.1	165.0	2.534
	**70 V**	163.5	163.9	165.6	166.3	168.7	169.6	166.3	2.479
	**80 V**	163.7	166.6	166.7	170.3	172.4	173.4	168.9	3.785
**ΔCCS (%)**	**10 V**	0.000	0.000	0.000	0.000	0.000	0.000	0.000	0.000
	**20 V**	-0.683	0.309	0.552	-0.061	-0.241	-0.537	-0.110	0.478
	**30 V**	0.124	0.928	0.123	-0.061	0.060	-0.119	0.176	0.381
	**40 V**	0.807	1.051	0.797	0.000	-0.120	0.119	0.442	0.499
	**50 V**	0.186	0.433	0.429	0.121	0.120	-0.358	0.155	0.289
	**60 V**	0.000	1.361	0.613	0.485	0.542	0.298	0.550	0.454
	**70 V**	1.553	1.361	1.533	0.849	1.565	1.193	1.342	0.282
	**80 V**	1.677	3.030	2.207	3.275	3.793	3.461	2.907	0.806
